# The medicolegal landscape through the lens of COVID-19: time for reform

**DOI:** 10.1177/0141076820974988

**Published:** 2020-11-19

**Authors:** Arpan R Mehta, Tamás Szakmany, Annie Sorbie

**Affiliations:** 1 Centre for Clinical Brain Sciences, Edinburgh bioQuarter, Edinburgh, EH16 4SB, UK; 2 Nuffield Department of Clinical Neurosciences, University of Oxford, John Radcliffe Hospital, Headington, Oxford, OX3 9DU, UK; 3 Critical Care Directorate, Aneurin Bevan University Health Board, 97644 Royal Gwent Hospital, Newport, NP20 3UB, UK; 4 Department of Anaesthesia, Intensive Care and Pain Medicine, Division of Population Medicine, Cardiff University, Cardiff, CF14 4XN, UK; 5 School of Law, University of Edinburgh, South Bridge, Edinburgh, EH8 9YL, UK

The COVID-19 pandemic has brought out the best of the health and social care workforce globally, as acknowledged by the public. But the clapping has now stopped. Over 50,000 people who tested positive for coronavirus in the UK have died, a tragic figure that is more than double the UK Government's early ‘best case scenario’ estimate. Each death represents a life lost too soon, leaving behind grieving family and friends. At the same time, doctors and other healthcare professionals are exhausted and anxious, fearing both the implications of a second wave, and possible repercussions from decisions made under the strain of the pandemic.

There has been polarised debate around whether doctors should be granted immunity from civil and criminal negligence claims and regulatory proceedings arising from treatment provided during COVID-19.^1,2^ Here, we argue that this focus on temporary statutory immunity is a distraction from pre-existing concerns that several aspects of the current medicolegal system are not fit for purpose – for doctors or for patients. Areas where there is no ‘quick fix’ include: the need for reform of the clinical negligence system; concerns in relation to regulatory proceedings; and the potential for BAME (black, Asian, and minority ethnic) doctors (and patients) to be disproportionately impacted in both areas. These issues are critical, since they each have a direct impact on multiple stakeholders, including on those who deliver and receive healthcare. However, there has been a tendency for these to be considered from single-minded viewpoints; accordingly, we aim in this paper to provide a more holistic view. Rather than pursuing immunity legislation, we say that the time is right for more comprehensive action, including an independent Public Inquiry to scrutinise these issues, taking into account all of the interests engaged (Figure 1).

**Figure 1.**
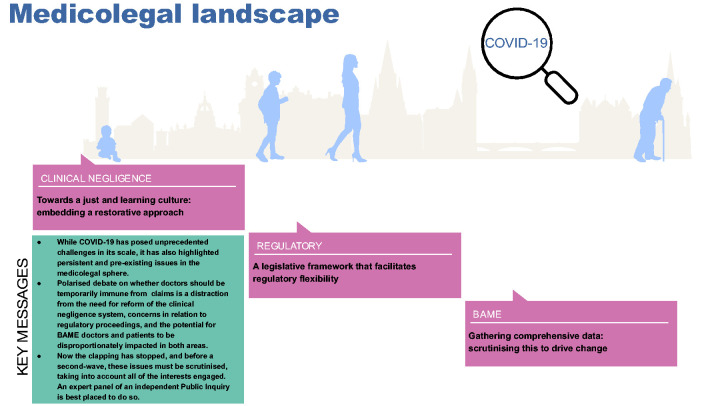
The medicolegal landscape through the lens of COVID-19. Reform needed in clinical negligence and regulatory spheres and an assessment of the medicolegal impact on BAME doctors and patients. BAME: black, Asian, and minority ethnic.

## Moving beyond immunity: the case for medical negligence reform

It bears repetition that patient safety issues are notoriously under-reported and, in most cases where a patient has suffered harm following a medical error, a claim in negligence is not pursued. In the minority of cases, doctors (and indeed other healthcare professionals) may find themselves exposed to liability for negligent medical treatment, but only when they have provided diagnoses or treatment that fall below the standard of a reasonable doctor, and where this has caused harm. This can be dealt with under criminal and/or civil law. While criminal cases are extremely rare, a recent review of gross negligence manslaughter, led by Sir Norman Williams, acknowledges both the effects of the unexpected death of a loved one for bereaved family and friends, and also the effect this may have on the medical team providing care.^3^ Recommendations from this review underpin the need for a just and learning culture in healthcare, where cases can be dealt with fairly, transparently and compassionately. It further highlights the disproportionate representation of BAME professionals within criminal proceedings, and calls for careful consideration to avoid perceived (and, by implication, actual) injustice.

Contrast this with recent discussions in the UK concerning whether doctors should be granted immunity from proceedings in respect of treatment provided during the COVID-19 pandemic, along the lines of emergency legislation introduced in some US states, including New York.^2^ This US legislation grants healthcare professionals, acting in good faith, temporary immunity from civil and criminal liability (but not wilful or intentional criminal harm, or reckless misconduct). However, it does not extend to protection against regulatory proceedings.^4^ The debate in the UK has focused in particular on immunity from clinical negligence claims.^1^ Those in favour point to the emotional and professional burden of dealing with a clinical negligence claim, regardless of the outcome, and even if doctors are protected financially by state-backed indemnity arrangements. Those to the contrary contend that immunity is superfluous and inappropriate, given the ability of the law as it stands to protect doctors from being unfairly judged, the extraordinary circumstances in which treatment has been provided and the need to consider, among other interests, those of patients. Both sides of the debate raise important points, but we suggest that, by narrowing the discussion to COVID-19-specific short-term statutory measures, this obscures three more pressing and persistent issues in the medicolegal landscape, the first of which is the unsatisfactory state of the clinical negligence system.

The need for clinical negligence reform has been mooted for over 30 years. COVID-19 is not the genesis of these issues; rather it has brought into sharp relief longstanding problems with the system. Concerns beyond the pandemic relate to the financial cost of clinical negligence claims, and the resulting impact on the availability of resources for healthcare, not only in monetary terms, but also in relation to the clinical and administrative burden this generates.^5^ A recent review of medical device safety also highlights the negative impact of current processes for redress following medical harm on patients, including financial hardship, family breakdowns, and loss of identity and self-worth.^6^ Administrative schemes that are limited to low-value claims, and directed more to cost control than addressing patient concerns, have thus far failed to deliver a ‘just redress scheme’,^7^ or a comprehensive alternative to the *status quo*. Real change will not come about through the ‘quick fix’ of temporary immunity, but rather through sustained, systems-level action to support staff, improve patient safety, and commitment to a just and learning culture in healthcare,^5^ as endorsed in the criminal context by the Williams review.^3^

NHS Resolution has recently highlighted a number of concrete examples of how this can be achieved in their 2019 report, *Being Fair*.^8^ In particular, this underlines the benefits of a restorative approach in the aftermath of incidents, which ‘holds people accountable by looking forward to what must be done to repair, to heal and to prevent’.^9^ This practice is not merely aspirational; it has been operationalised by Mersey Care, an NHS Foundation Trust providing mental health services, to deliver a transformative shift from a culture of blame to one of trust and learning. This initiative, and others, which stem from a collaborative approach, underline the interconnectedness of patient safety and staff wellbeing, and the need to engage with multiple perspectives to drive real change.

## Regulatory proceedings

Our second concern is that an exclusive focus on immunity legislation also narrows consideration of medicolegal issues to civil claims for medical negligence, when, in fact, many doctors are more worried about the prospect of a complaint to the General Medical Council in relation to their fitness to practise. This is outwith the scope of the US immunity legislation referred to above. In the UK, this is a particularly thorny issue in the medical community, given pre-existing unease concerning the individualisation of systemic failures, in particular following the criminal and regulatory proceedings pursued against Mr David Sellu and Dr Hadiza Bawa-Garba, among others.

During the pandemic, the position has remained that the General Medical Council's professional guidance applies. However, healthcare professionals' anxieties have not gone unheeded; the chief executives of the health and social care regulators issued an early joint statement on how they will regulate in light of COVID-19.^10^ Many have welcomed recognition from their regulatory body of the challenging circumstances in which they are operating, and of the flexibility that the pandemic response has demanded, including working in unfamiliar surroundings or being redeployed to different clinical areas.^11^

So where does this leave doctors who are concerned about fitness to practise proceedings? Guidance from the British Medical Association indicates that complaints are unlikely when decisions are reasonable, based on the best available evidence, informed by guidance, collaborative and, as far as possible, promote effective and safe care.^12^ We further welcome the joined-up approach of the regulators which, in our view, points to an acknowledgement that the pandemic response is a ‘team effort’. As noted in the General Medical Council's own guidance about prioritising access to treatment, difficult decisions should not, as far as is practicable, fall on one clinician's shoulders. Most recently, the General Medical Council has issued specific guidance on how it will assess the risk to public protection posed by a doctor as a result of concerns about their practice during the pandemic.^13^ This will sit alongside existing guidance and processes in order to inform the work of General Medical Council staff who look into fitness to practise concerns on a case-by-case basis.

But what other steps could be taken as we move forward, and as memories of recent working conditions dull? The exclusion of regulatory action from the emergency immunity legislation enacted in some US states was unlikely to have been an oversight. To preclude patients from making any complaint in relation to the pandemic to a body charged with their protection is a draconian measure and, in the UK, incompatible with the General Medical Council's statutory objective and functions. The role of ongoing and responsive engagement by the General Medical Council – with doctors, patients and other stakeholders – is vital to maintain the public’s and professionals’ trust in the regulatory system. Organisational memory is a key part of creating a just culture in healthcare, which, in turn, has been shown to improve patient safety. As such, we do not characterise this task as a ‘balance’ to be struck between supporting doctors, on the one hand, and protecting patients on the other, with the implication that promoting one interest necessarily diminishes the other. The porosity of these roles has been laid bare by the pandemic, where caregivers have become patients, and members of the public have stepped up to support the delivery of NHS services. Here, too, we would also urge consideration of the bigger picture; it is more essential than ever that the regulatory system itself is fit for purpose. Over six years ago, the Law Commission's report outlined sweeping legislative changes to the regulation of health and social care professionals in the UK, to fix a legislative framework that is ‘cumbersome and inflexible’.^14^ The Government's response promised to make changes that will facilitate more efficient and responsive fitness to practise procedures, which better support professionals and make regulation more responsive and accountable,^15^ though with no clear timetable for delivery. The pandemic has further underlined the importance of a regulatory framework that promotes, rather than constrains, regulatory flexibility, not only for doctors, but for all registered healthcare professionals.

## Impact on BAME doctors and patients

Finally, our third concern is that discussions that focus on immunity overlook the disproportionate impact of the pandemic on BAME doctors and patients. Despite early rhetoric, COVID-19 is a disease that *does* discriminate.^16^

BAME doctors have suffered greater morbidity and mortality, with evidence that they have been more exposed to consequences of inadequacy of personal protective equipment.^17^ Prior to the pandemic, BAME healthcare professionals were already disproportionately represented across the medicolegal landscape, reflected in levels of disciplinary action against BAME staff groups,^8^ higher rates of referrals of doctors to their regulator,^18^ and disproportionate representation in criminal proceedings.^3^ Research commissioned by the General Medical Council prior to the pandemic suggests that factors that help explain higher rates of referrals include matters such as inadequate induction and blame cultures, where doctors who are ‘outsiders’ are at particular risk.^18^ In promoting its recent guidance, the General Medical Council should be commended for its recognition both of ‘the disproportionate impact of disease and mortality rates on individuals from black and minority ethnic (BME) backgrounds’ and differences in referral patterns between BAME and white doctors (paragraph 7).^13^ During the first wave of COVID-19, doctors have worked in unfamiliar surroundings, outside of their usual clinical areas, and we are also of the view that there is clear potential for the factors which drive higher referral rates to be exacerbated in this context. However, as well as careful consideration of individual cases, we also call for scrutiny of the wider cohort of cases over this period to identify if there are broader patterns or learning points that can be swiftly identified and disseminated – both to regulators and across the health ecosystem – as we move into a second wave of the virus.

On the other hand, it is now established that BAME patients are also disproportionately affected by the virus, with demonstrably greater risk of COVID mortality.^19^ Therefore, it must be kept in mind that any measures to restrict access to redress, be this civil or regulatory, may also disproportionately impact upon these groups of patients. Furthermore, COVID-19 has highlighted the need to conduct health research in a way that serves and represents the whole of our community.^20^ If we are to properly monitor and understand the medicolegal implications of the pandemic, for doctors and for patients, we believe these commitments apply equally to data collection and research in this area. The General Medical Council has already made decisive moves towards making the data it collects accessible, including in relation to fitness to practise procedures. We call for further progress to enhance data collection on incidents and ethnicity across the health and regulatory landscape, relating both to patients and to healthcare professionals. The issues we have discussed in this paper are both persistent and multi-faceted. If they are to be effectively addressed, this information will require careful quantitative and qualitative scrutiny as part of a coherent and collaborative research agenda. The General Medical Council's BAME Doctors' Forum is well placed to coordinate and inform the further work that is needed to progress this.

## Moving forward

COVID-19 is a disease which has not only posed unprecedented challenges in its scale, it has also highlighted and exacerbated pre-existing issues in the medicolegal sphere. While the focus in this domain has so far been on protecting doctors, through immunity legislation, our analysis indicates that meaningful action in this area, which promotes a just culture in healthcare, demands engagement with a more diverse range of interests. Thus far, the medicolegal issues we have raised have been touched on at a meeting convened by the all-party parliamentary group (APPG), dedicated to ensuring that lessons are learned from the UK’s handling of COVID-19. Public Health England's review into the impact of COVID-19 on ethnic minorities alludes to action being required to change workplace environments,^21^ but does not specifically address the potential medicolegal consequences for doctors or patients that we have identified. Leaders have indicated that this report has failed ethnic minorities and we now consider that an independent Public Inquiry,^22^ with a series of expert panels addressing specific issues, must be convened to address these cross-cutting concerns in the context of COVID-19. However, we also call for more comprehensive action to address the persistent issues revealed by the lens of COVID-19, including embedding a restorative approach in healthcare, ensuring that regulatory flexibility is supported by a coherent legislative framework and gathering comprehensive data to facilitate research and drive change (Figure 1).

## Declarations

**Ethics approval:** Not applicable.

**Guarantors:** ARM and AS.

**Contributorship:** This article was conceived by ARM. All authors contributed to the article's development and approved the final manuscript.
